# Exploring Spirituality, Loneliness and HRQoL In Hispanic Cancer Caregivers

**DOI:** 10.1093/geroni/igab046.2578

**Published:** 2021-12-17

**Authors:** Jennifer King, Terry Badger, Chris Segrin, Cynthia Thomson

**Affiliations:** 1 University of Arizona Cancer Center, Marana, Arizona, United States; 2 University of Arizona, Tucson, Arizona, United States; 3 University of Arizona, Mel and Enid Zuckerman College of Public Health, Tucson, Arizona, United States

## Abstract

Providing care to an aging society in the new normal requires increased attention to the informal caregivers who support the health and well-being of older adults with chronic conditions. Hispanic caregivers carry a high caregiver-associated burden. Health disparities experienced by Hispanics, coupled with the emotional, social and physical demands of caregiving, may set an unprecedented risk for lower health-related quality of life (HRQoL). In a quantitative analysis, we investigated the relationship between spirituality, loneliness and HRQoL in Hispanic cancer caregivers using baseline data from the Support for Latinas with Breast Cancer study (N= 234 Hispanic caregivers). Findings suggested an indirect effect of spirituality on HRQoL through reduced loneliness among more spiritual caregivers, effects that were independent of age. The second study was conducted using qualitative semi-structured interviews (N= 10) with Hispanic caregivers. Interviews evaluated spirituality and HRQoL in Hispanic cancer caregivers who reported variable levels of loneliness. Five themes emerged: caregiver experience, coping strategies, loneliness, religion to gain strength or support, and spirituality to gain strength or support. Results supported the role of spirituality in promoting higher HRQoL in Hispanic cancer caregivers and elucidated pathways to intervene on HRQoL through spirituality. With Hispanics often underutilizing formal services, having an improved understanding of caregiving experiences, particularly related to spirituality, will support the development of culturally-relevant strategies and programming to promote HRQoL for Hispanic caregivers.

